# Luminophore Application Study of Polymer-Ceramic Pressure-Sensitive Paint

**DOI:** 10.3390/s130607053

**Published:** 2013-05-29

**Authors:** Hirotaka Sakaue, Tatsunori Hayashi, Hitoshi Ishikawa

**Affiliations:** 1 Institute of Aeronautical Technology, JAXA, Chofu, Tokyo 182-8522, Japan; 2 Department of Mechanical Engineering, Tokyo University of Science, Chiyoda, Tokyo 102-0073, Japan; E-Mails: advmeas@chofu.jaxa.jp (T.H.); ishi@rs.kagu.tus.ac.jp (H.I.)

**Keywords:** pressure-sensitive paint, polymer ceramic, luminophore application

## Abstract

A polymer-ceramic pressure-sensitive paint (PC-PSP) is a fast responding and sprayable PSP which has been applied for capturing global unsteady flows. The luminophore application process is studied to enhance the characterization of the PC-PSP. A dipping deposition method is used to apply a luminophore on a polymer-ceramic coating. The method selects a solvent by its polarity index. The characterization includes the signal level, pressure sensitivity, temperature dependency, and response time. It is found that the luminophore application process affects the steady-state characterizations, such as the signal level, pressure sensitivity, and temperature dependency. A range of change for each characterization, which is based on the minimum quantity, is a factor of 4.7, 9, and 3.8, respectively. A response time on the order of ten microseconds is shown. The application process is not a dominant factor for changing the response time, which is within the uncertainty of the thickness variation. Comparisons of the effects on the luminophore application process and the polymer content are made to discuss the PC-PSP characterization results.

## 1. Introduction

A luminescent pressure-sensitive paint (PSP) sensor combined with a fast frame-rate camera has been used for capturing global unsteady flows and for short duration testing [1–3]. The PSP uses a photophysical oxygen quenching process to relate an oxygen pressure of a testing fluid to a luminescent signal. It is composed of a luminophore and a supporting matrix. The former gives a luminescent signal and the latter holds the luminophore on a testing article. By using a porous material as a supporting matrix, this type of PSP (porous PSP) can increase the mass diffusion inside the pore to enhance the response time of the PSP and the response time is on the order of ten microseconds [4]. Even though its response is fast, the porous PSP has a material limitation. For example, anodized-aluminum pressure-sensitive paint (AA-PSP), which is one of the porous PSPs, is only applicable to aluminum [5]. This limits the application of a porous PSP to testing articles with a limited material range.

**Figure 1.**
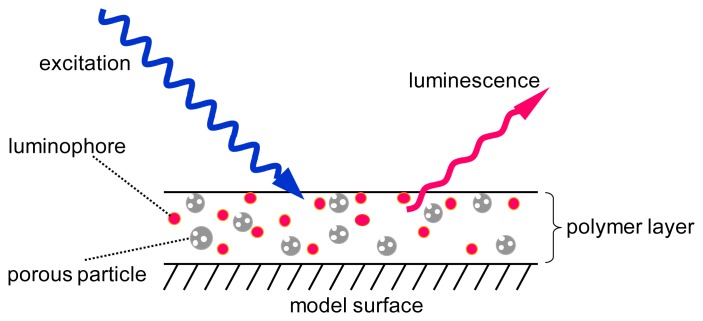
Schematic description of a polymer-ceramic PSP (PC-PSP).

Recent progress in the fast responding PSP area are the development of a polymer-ceramic PSP (PC-PSP) [6–10]. The supporting matrix of this PSP is composed of a porous particle and a polymer (Figure 1). The former enhances the response time, while the latter provides the spraying ability. Because two components are used as the supporting matrix, the polymer content, which is a ratio of a polymer to a porous material, is an additional parameter that affects the characterization of the PC-PSP. Sakaue *et al*. reported that the characterization results were dependent on the polymer content and the most influencing characteristic was the response time. By varying the polymer content from 10 to 90 w%, the response time was changed by five orders of magnitude [10].

For a porous PSP, the luminophore application process was one of the parameters used to control the PSP characterization [5,11,12]. It was reported that the signal level, pressure sensitivity, and temperature dependency were dependent on the luminophore application process. Because a PC-PSP uses a porous material as one of the supporting matrix components, the application process would also be a parameter to control the PSP characterization features, such as the signal level, pressure sensitivity, temperature dependency, and the response time. Based on Sakaue, the dipping deposition method was used to apply the luminophore onto a polymer-ceramic coating [5]. The polarity index of the solvent for the deposition method is a parameter to study this luminophore application process. Comparisons were made to discuss the effects of the polymer content [10] and the application process on the PSP characterization results.

## 2. Experiments and Methods

### 2.1. Materials

We chose a silica gel from Sigma-Aldrich (St. Louis, MO) as a porous particle. It has a mean particle size of 2 to 25 μm. We chose RTV from Shinetsu Silicone (Tokyo, Japan) as a polymer. To mix these components, we used dichloromethane as a solvent. The polymer-particle mixture was ultrasonicated for 20 min to reduce the aggregation of the particles, then it was spin-coated on a 20-mm square aluminum plate. We adjusted the thickness of the polymer-ceramic coating as 10 ± 3 μm, which was measured by an eddy current apparatus (LZ-300, Kett, Tokyo, Japan). The polymer to particle ratio (polymer content) was 20 w%.

**Figure 2.**
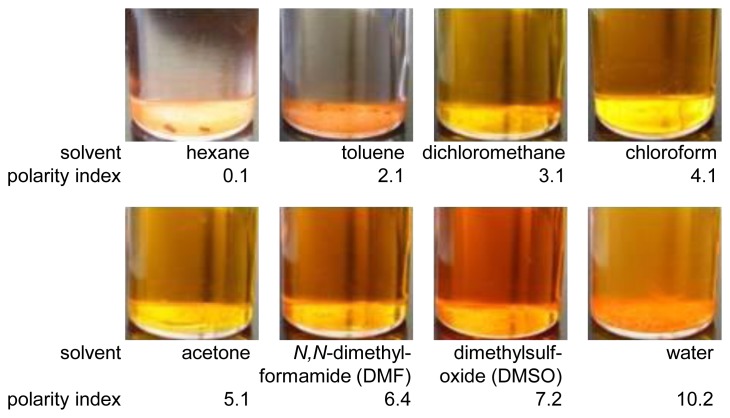
List of dipping solvents and photographs of bathophen ruthenium in these solvents.

We used bathophen ruthenium from GFS Chemicals (Powell, OH, USA) as a luminophore. Based on Sakaue, dipping solvents were selected based on their polarity index [5]. Total eight solvents were chosen. As shown in Figure 2, we can see the solubility of bathophen ruthenium varied according to the solvent used. Bathophen ruthenium was completely dissolved in the solvents that ranged from 3.1 to 7.2 on the polarity index. At lower polarity indexes (0.1 and 2.1) and the highest index of 10.2, the luminophore was partially dissolved. We weighed bathophen ruthenium to be 0.1 mM in concentration if the luminophore was completely dissolved.

**Figure 3.**
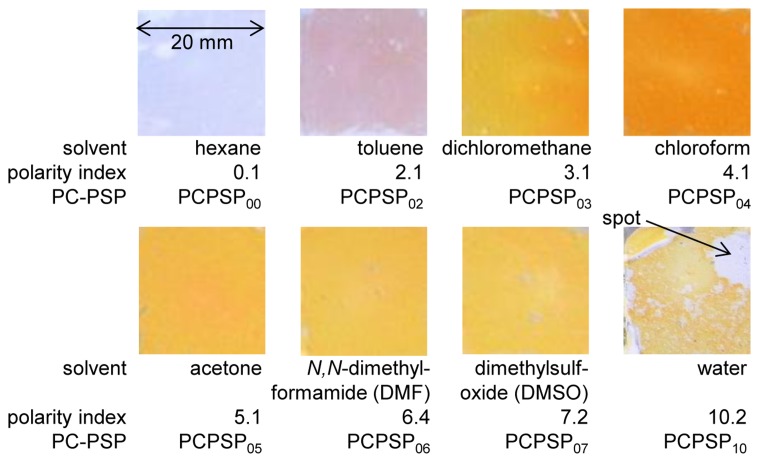
Photographs and identification of PC-PSPs.

For each dipping solution, three PSP samples were prepared to study the repeatability of the PSP preparation. The polymer-ceramic coating created was dipped in each solution or mixture for 20 min at room conditions. Figure 3 shows photographs of the PC-PSPs. The developed PC-PSPs were identified by the solvents used, which are listed in Figure 3. We can see that the luminophore application by the dipping deposition method varied according to the solvent used. The luminophore indicated by its orange color was most applied for PCPSP_04_, and less applied for PCPSP_00_ and PCPSP_02_. We can see a non-uniform spot for PCPSP_10_. Because of the effects of mixtures, the luminophore was applied in spots for PCPSP_10_. PC-PSP dipped in dichloromethane, which was the same solvent used to create a PC-PSP in the previous study, was used as a reference PC-PSP [10]. The luminescent outputs from all the samples can be quantified as the luminescent intensity, which is discussed in Section 2.2.

### 2.2. Steady-State Characterization

**Figure 4.**
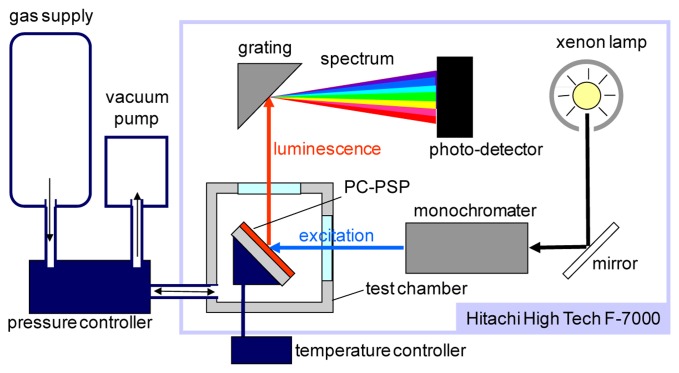
Schematic of the PC-PSP calibration setup.

Figure 4 schematically describes the steady-state characterization setup, which consists of a F-7000 spectrometer (Hitachi High Technologies, Tokyo, Japan) and a pressure- and temperature-controlled chamber. This system obtains the luminescent spectrum of a PC-PSP with varying pressures and temperatures. We characterized the signal level, pressure sensitivity, and temperature dependency from this setup. The excitation wavelength was set at 460 nm. The luminescent intensity of a PC-PSP was determined by the integration of the spectrum within 600 to 700 nm. The test gas was dry air. Throughout our characterizations, the reference conditions were 100 kPa and 25 °C.

For the signal-level characterization, all the PC-PSP samples were measured with the same optical setup in the system but replacing samples in the chamber under the reference conditions. Based on Liu *et al.*, the luminescent intensity, *I*, can be described by the gain of the photodetector in a spectrometer, *G*, the emission from PC-PSP, *I_PCPSP_*, the excitation in the spectrometer, *I_ex_*, and the measurement setup component, *f_set_* [13]. Here, *f_set_* covers the factors affecting to the transfer function of a light path within the measurement setup:

(1) $$I=GI_{\text{PCPSP}}I_{ex}f_{\text{set}}$$

In our setup, *G*, *I_ex_*, and *f_set_* were the same for all PC-PSP samples. We non-dimensionalized *I* by that of PCPSP_03_. We call this value as the signal level, *η*, shown in Equation (2):

(2) $$\eta =\frac{I}{I_{\text{PCPSP}03}}(\%)$$

For the pressure calibration, the pressure, *P*, in the chamber was set from 5 to 120 kPa at a constant temperature of 25 °C. The luminescent intensity at the reference conditions, *Iref*, was used to derive *Iref*/*I*. This quantity can be related to pressures using the Stern-Volmer relationship [14]:

(3) $$\frac{I_{\text{ref}}}{I}=A_{P}+B_{P}.P$$

where *A_P_* and *B_P_* are calibration constants. A PSP with porous surface would show a non-linear relationship due to an oxygen adsorption on the porous surface [5]. Because a PC-PSP is a combination of a porous material and a polymer, the same model describing a porous PSP would not be physically correct. As an alternative, we used the second-order polynomial to modify Equation (3):

(4) $$\frac{I_{\text{ref}}}{I}=A_{P}+B_{P}.P+C_{P}.P^{2}$$

where *C_P_* is an additional calibration constant.

The pressure sensitivity, *σ*, describes the change in *I* over a given pressure change. A high *σ* is more sensitive to the pressure. This corresponds to a slope of Equation (4) at the reference conditions:

(5) $${\sigma =\frac{d(I_{\text{ref}}/I)}{dp}|}_{P=P_{\text{ref}}}=B_{P}+2C_{P}.P_{\text{ref}}\;(\%/\text{kPa})$$

A PSP, in general, has a temperature dependency [14]. This influences *I*, which can be described as the second-order polynomial in Equation (6):

(6) $$\frac{I}{I_{\text{ref}}}=A_{T}+B_{T}.T+C_{T}.T^{2}$$

where A_T_, B_T_, and C_T_ are calibration constants. For the temperature calibration, the temperature, T, was set from 10 to 50 °C with a constant pressure at 100 kPa.

We defined the temperature dependency, *δ*, which is a slope of the temperature calibration at the reference conditions (Equation (7)). If the absolute value of *δ* is large, it tells us that the change in *I* over a given temperature change is also large. This is unfavorable condition for a pressure sensor. On the contrary, zero *δ* means that PC-PSP is temperature independent:

(7) $${\delta =\frac{d(I/I_{\text{ref}}\;)}{dT}|}_{T=T_{\text{ref}}}=B_{T}+2C_{T}.T_{\text{ref}}(\%/{}^{\circ}\text{C})$$

### 2.3. Unsteady-State Characterization

**Figure 5.**
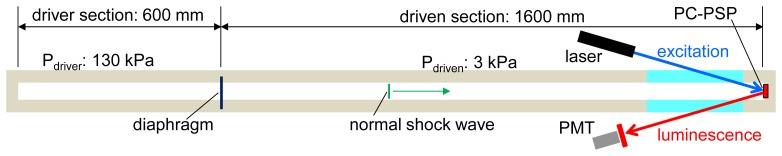
Schematic of the shock tube setup.

Figure 5 shows a schematic of a shock tube setup to determine the response time for the unsteady-state characterization. A cross-sectional area was 30-mm square, and the driven and driver lengths were 1,600 and 600 mm, respectively. The test gas was air at the temperature of 293 K. When the diaphragm between the driver and the driven sections was ruptured, a planar shock wave propagates into the driven section. We set the driver and driven pressures as 130 and 3 kPa, respectively, that created a planar shock wave with the Mach number of 2.08. A PC-PSP was placed on the end wall, which was illuminated through an acrylic window using a BLM-7000-H08D, 7W 465-nm laser (Sumitomodenko, Osaka, Japan). The laser illuminated the PC-PSP from one side of the window, and the luminescent intensity, *I*, from the PC-PSP was measured from the other side of the window. To characterize the response time on the order of ten to hundred microseconds, we used a H57730-04 photomultiplier tube (PMT, Hamamatsu Photonics, Shizuoka, Japan) combined with a band-pass filter of 650 ± 50 nm to acquire *I*. It satisfies our need to acquire *I* on the order of mega-samples per second, which was fast enough to resolve the response time of PC-PSP. Because the PC-PSP was mounted on the end wall, the change of *I* was only due to the pressure impact by the normal shock.

The PC-PSP on the end wall was initially under the driven pressure, *P_driven_*. Once the shock wave impacted on the end plate, the PSP experienced a sudden pressure jump, *P_reflect_*. The luminescent intensity, *I*, was converted to pressures using Equation (4). To extract the time delay, the pressure, *P*, was normalized, *P_norm_*:

(8) $$P_{\text{norm}}=\frac{P-P_{\text{driven}}}{P_{\text{reflect}}-P_{\text{driven}}}$$

The response time of the PC-PSP, *τ*, was defined as a time delay from a step change of pressure, which was the time duration approaching 90% change of *P_norm_* [5].

## 3. Results and Discussion

### 3.1. PC-PSP Spectrum

**Figure 6.**
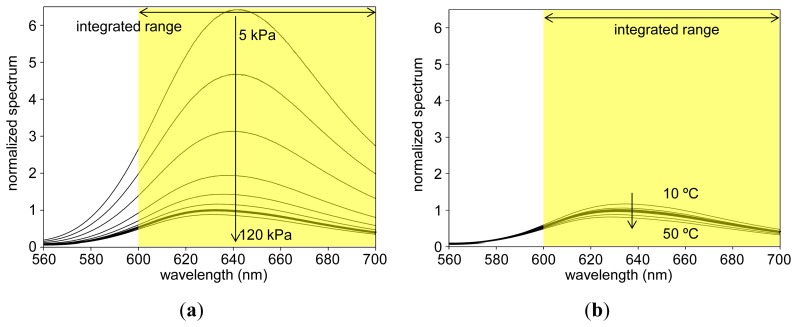
**(a)** Pressure spectra and **(b)** temperature spectra of PCPSP_03_. Thick line shows the spectrum at the reference conditions of 100 kPa and 25 °C.

Figure 6(a,b) show the luminescent spectra of PCPSP_03_ with varying pressures and temperatures, respectively. Spectra were normalized by the luminescent peak at the reference conditions. We can see that with increase of the pressure, the luminescent spectrum decreased due to the oxygen quenching [14]. With the increase of the temperature, we can see the spectrum decreased due to the thermal quenching [14]. We can see that a luminescent peak existed around 640 nm. As described in Section 2.2, we integrated an obtained spectrum from 600 to 700 nm to determine the luminescent intensity, *I*, for a given pressure and temperature.

### 3.2. Signal Level

**Figure 7.**
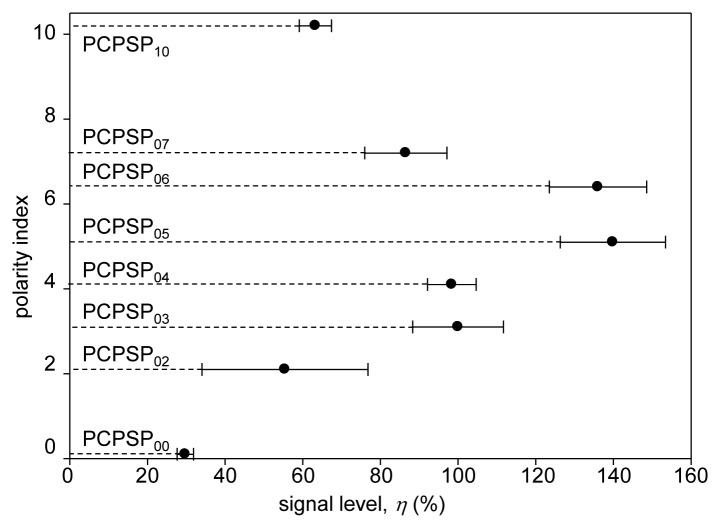
Relationship between the signal level, *η* (%), and the polarity index.

The signal level, *η*, was determined from Equation (2), which was shown in Figure 7. We can see that *η* was related to the polarity index of the luminophore application process. If we compare the mean value of *η*, there was a peak that gave the maximum *η*. It would lie between 4 and 6 of the polarity index. For the present experiment the maximum *η* was obtained at the polarity index of 5.1 from acetone as a dipping solvent. A range of change was a factor of 4.7, which was based on the minimum *η*. In the previous study, dichloromethane was used as a dipping solvent for the luminophore application process [10]. We found that acetone as a dipping solvent enhanced *η* by 40%. By varying the polymer content in the previous study, *η* was varied more than a factor of two [10]. Based on the present results obtained, we found that the luminophore application process effected more than the polymer content to control *η*.

Based on the photographs of PC-PSPs (Figure 3), PCPSP_04_ was the most applied luminophore. However, it did not give the maximum *η*. The reduction of *η* would be due to the concentration quenching [15]. As previously reported, the concentration as well as the dipping duration would be other factors to control *η* [11,12]. Due to a ±30% variation in the PC-PSP thickness that directly related to the surface area for applying the amount of luminophore, we saw relatively large errors. Table 1 summarizes the PC-PSP characterizations of the present results, as well as the comparisons from the previous study, which controlled the polymer content [10].

### 3.3. Pressure Sensitivity

**Figure 8.**
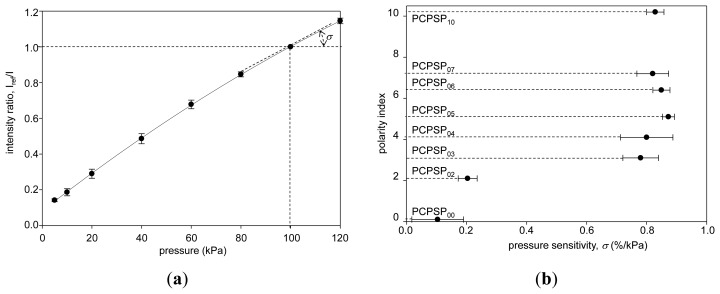
(**a**) The pressure calibration of PCPSP_03_. (**b**) Relationship between the pressure sensitivity, *σ*(%/kPa), and the polarity index.

Figure 8(a) shows the pressure calibration of PCPSP_03_. Calibration plots were fitted with Equation (4). A fairly linear relationship was seen. Even though a large error was seen in the signal level (Section 3.2), the ratio with the reference signal, *I_ref_/I*, showed a smaller error. Figure 8(b) shows the pressure sensitivity, *σ*, related to the polarity index. It ranged from 0.1 to 0.9%/kPa. There were two groups: PC-PSPs with higher *σ*(over 0.6%/kPa) and lower *σ*(less than 0.3%/kPa). PC-PSPs with the polarity index above 3.1 were the former group, while PC-PSPs with the polarity index below 2.1 were in the latter. Based on the photographs of the PC-PSPs (Figure 3), the luminophore was less applied for the latter group. It was seen from the mean *η*, which was less than 40% from this group (Figure 7). Even though PCPSP_10_ was the same level of *η* to that of PCPSP_02_, the luminescent intensity, *I*, was averaged including non-luminophore applied spots. The luminophore-applied area would be sensitive to the pressure that resulted in a high *σ* of 0.8%/kPa. In the previous study, the polymer content was not a dominant factor to control *σ* [10]. On the contrary, the luminophore application process was one of the dominant factors to control *σ*.The results are summarized in Table 1, along with the comparisons of the previous study [10].

### 3.4. Temperature Dependency

**Figure 9.**
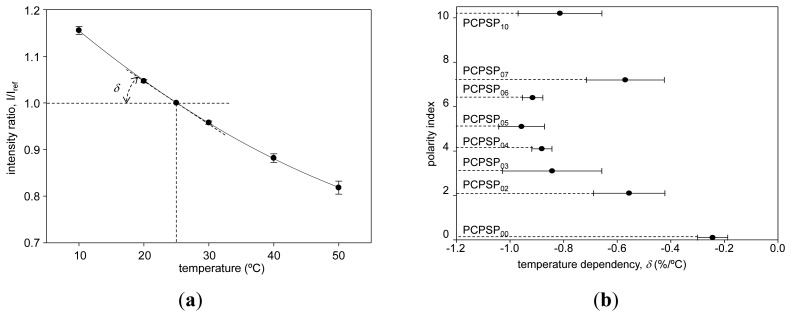
(**a**) The temperature calibration of PCPSP_03_. (**b**) Relationship between the temperature dependency, *δ*(%/°C), and the polarity index.

Figure 9(a) shows the temperature calibration of PCPSP_03_. Calibration plots were fitted with Equation (6). The calibrations showed a monotonic decrease in *I* with increase of the temperature. Similar to the pressure calibration, the ratio with the reference signal, *I/I_ref_*, showed a smaller error than the error bar of the signal level (Section 3.2). Figure 9(b) shows the temperature dependency, *δ*, related to the polarity index. As a general trend, the absolute value of *δ* showed the maximum around the polarity index of 5. As a pressure sensor, *δ* is an undesirable quantity; zero *δ* is desirable. The results showed that the least temperature dependent PSP can be obtained by PCPSP_00_ whose polarity index was 0.1 from hexane. In the previous study, there was a factor of two differences in *δ* by the polymer content [10]. In the present results, *δ* was varied by a factor of 3.8. Compare to the effect on the polymer content, *δ* was affected more by the luminophore application process (Table 1).

### 3.5. Response Time

**Figure 10.**
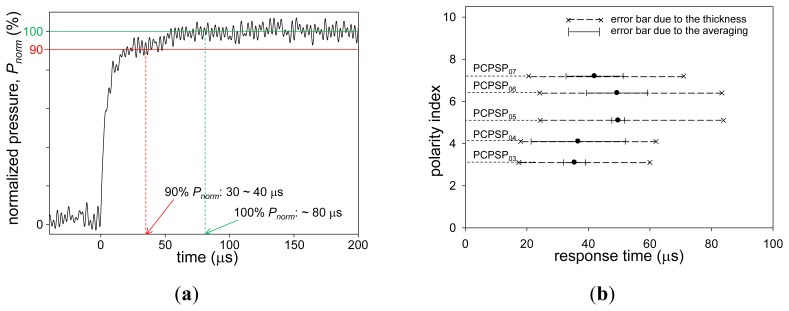
(**a**) Step pressure response of PCPSP_03_. (**b**) Relationship between the response time, *τ*(μs), and the polarity index.

Figure 10(a) shows a step response of PCPSP_03_ following a normal shock impact on the end plate. The step response was shown as *P_norm_* related to the measurement time. Here, *P_norm_* was determined from Equation (8). We can see a fast and slow component in the response. A fast component could be seen till 80% of *P_norm_*, which was around 10 μs from the shock impact. A slow component could be seen from 80% to 100% of *P_norm_*. The value of *τ*, which was determined by a time delay approaching a 90% change in *P_norm_*, was around 30 to 40 μs. If *P_norm_* approached to 100%, PCPSP_03_ needed around 80 μs.

Figure 10(b) shows *τ* related to the polarity index. A single-shot measurement was required to characterize *τ*. Due to a low *η*, we could not characterize *τ* of PCPSP_00_, PCPSP_02_, and PCPSP_10_. For each PC-PSP sample, there was a variation in the coating thickness by ± 30%. Referring from Kameda *et al.*, we included the error bar as the squared value of the thickness uncertainty, which was from 49% to 169% of the determined *τ* [4]. They used the dipping procedure, which was the same luminophore application process, to apply the luminophore onto a porous supporting matrix of anodized aluminum. On the other hand, Sugimoto *et al.* reported that the response time was independent of the PC-PSP thickness [16]. They sprayed the luminophore onto the polymer-ceramic coating, while the present case used the dipping process. The latter would create the luminophore-applied layer instead of the luminophore attached on the coating surface. Further experimental studies would give us clear understanding of the relationship between the response time and the PC-PSP thickness.

In the present study, to discuss the response time error, we followed Kameda *et al.*, which used the same luminophore application process. Three PC-PSP samples for each luminophore application process were prepared and tested to characterize *τ*, which was shown as an averaged value with its standard deviation as an error bar (Figure 10(b)). We can see that there was a variation in *τ* by the PC-PSP samples as well. If we consider these uncertainties, the thickness uncertainty was dominant; all *τ* was within the thickness uncertainty. If we roughly determine *τ* from the averaged values, it ranged from 36 to 50 μs. If we consider the thickness uncertainty, *τ* can be said to be on the order of ten microseconds. The luminophore application process did not greatly influence to *τ*, which was within the thickness uncertainty. On the contrary, the polymer content was one of the dominant factors to control *τ*[10]. The difference of five orders of magnitude was shown (Table 1).

## 4. Conclusions

The luminophore application process of polymer-ceramic pressure-sensitive paint (PC-PSP) was studied to enhance its characterization for capturing global unsteady flows. A dipping deposition method was used to apply the luminophore onto a polymer-ceramic coating, which varied according to the polarity index of the solvents used for dipping. Bathophen ruthenium was used as a luminophore, and the polymer-ceramic coating contained the polymer content of 20 w% with its thickness of 10 ± 3 μm. Comparisons were made between the PC-PSPs with varied polymer content the same luminophore and the coating thickness [10]. It was found that the luminophore application process affected to the steady-state characterization features of the PC-PSPs, such as the signal level, pressure sensitivity, and temperature dependency. The most affected characterization was the pressure sensitivity, which changed by a factor of nine. The signal level changed by a factor of 4.7. These were more affected by the luminophore application process than by the polymer content. The temperature dependency showed a larger change due to the luminophore application process than to the polymer content. Even though there was a change in the response time by five orders of magnitude due to the polymer content, the luminophore application process showed a minimal effect, which was within the thickness uncertainty.

## Figures and Tables

**Table 1.** Summary of PC-PSP characterizations as well as the comparisons from the previous study that discussed the polymer content [10].

**PC-PSP Characterizations**	Luminophore Application *1	Polymer Content [10] *2	**Range of Change based on the Minimum Quantity**
Signal level, *η*(%)	30∼140	35∼100	*1: Factor of 4.7 *2: Factor of 2.9
Pressure sensitivity, *σ*(%/kPa)	0.1∼0.9	0.8∼0.9	*1: Factor of 9 *2: Factor of 1.1
Temperature dependency, *δ*(%/°C)	−0.25∼−0.95	−0.65∼−1.35	*1: Factor of 3.8 *2: Factor of 2.1
Response time, *τ*(μs)	36∼50	300∼10,000,000	*1: Same order of magnitude *2: Order of 5
